# Performance of high-level Spanish athletes in the Olympic Games according to gender

**DOI:** 10.1371/journal.pone.0251267

**Published:** 2021-05-06

**Authors:** Alejandro Leiva-Arcas, Raquel Vaquero-Cristóbal, Lucía Abenza-Cano, Antonio Sánchez-Pato

**Affiliations:** Olympic Studies Center, Faculty of Sport, Catholic University of Murcia, Murcia, Spain; Universidad de Almería, SPAIN

## Abstract

No studies have been found that analyzed the probabilities of high-level athletes according to gender for accessing programs that promote the professionalization of sports, and participation and success in the OG in Spain. This could explain the gender differences in these parameters and the trend towards more egalitarian data in recent years. The objective of this study was to analyze the probabilities of Spanish high-level athletes for participating and achieving sporting success in the 2008, 2012 and 2016 Olympic Games (OG). Data relating to a sample of 3757 high-level Spanish athletes (2398 men and 1359 women) between 2005 and 2016 were examined. The variables of gender, having obtained a scholarship from the Association of Olympic Athletes (ADO) program, training in a High Performance Center (CAR), participation and performance in the OG were analyzed. It was found that high-level female athletes were more likely than male athletes to belong to the ADO program (χ^2^ = 26,151; r* = 0.083; p = 0,000) and CAR (χ^2^ = 13,847; r* = 0.061; p = 0,000), and to qualify for an OG (χ^2^ = 22,838; r* = 0,078; p = 0,000), the same trend was found in the three Olympic cycles analyzed. With respect to the results in the OG, in general, no differences were found according to gender, although women were more likely to be finalists (χ^2^ = 4,406; r* = 0.071; p = 0,036), and more prominently in the 2016 OG (16.118; r* = 0.228; p = 0.000). The same applies to winning a medal (χ^2^ = 5.939; r* = 0.145; p = 0.015), more specifically bronze at the 2012 OG (χ^2^ = 6.215; r* = 0.149; p = 0.013). In conclusion, high-level female athletes in Spain have a higher percentage of access to high-level athlete support programs such as ADO and CAR, as well as participation in OG.

## Introduction

The modern Olympic movement was originally founded on the pillars of sporting amateurism. Its main advocate, Pierre de Coubertin, argued that the practice of sports should be dissociated from economic profit, which led to the exclusion of professional athletes from participation in the Olympic Games (OG) during the first decades of the twentieth century. This thinking was made official in Rule 26 of the Olympic Charter [1]. It was not until 1974 when the president of the International Olympic Committee (IOC), Lord Michael M. Killanin (1972–1980), made an amendment to Rule 26 (By-Law Rule) modifying the eligibility criteria for Olympic athletes. Under this By-Law Rule, athletes were allowed to obtain economic and material rewards derived from their sporting performance. According to the same amendment, effective control over the selection criteria for athletes was transferred to the International Federations (IF), causing certain IF to create specific incentives aimed at the full-professionalization of their athletes and high performance [2]. Finally, in 1986, IOC’s President Juan Antonio Samaranch (1980–2001) made a new amendment allowing Olympic athletes to sign lucrative advertising contracts while declaring the OG open to all athletes, whether amateur or professional [3].

This phenomenon has led to a restructuring of the goals and objectives of sports structures in order to meet the needs of professional athletes to optimize their sports performance [4, 5]. In this regard, the professionalization of sports has provided sufficient resources for its optimization through improvements in equipment, techniques and tactics, professionalization of agents, new training methods, more efficient analysis tools, etc. [6, 7]. As a result, the characteristics of the athletes have evolved, leading to a morphological optimization, especially in those skills that are most closely related to sports performance [8–10]. As a consequence, numerous research studies have addressed the importance of sports professionalization on sports performance [11–13]. Thus, an association has been found between physical abilities, anthropometric characteristics, physiological and neuromuscular aspects [14], and the probability of athletic success in Olympic sports, which are also influenced by other aspects such as previous experience and age [15–17].

In order to seek better performance in elite sport, most nations have developed, to a greater or lesser extent, structures designed to promote sporting talent. In other cases, the promotion of high-level sport has been mostly based on college sport, as in the USA [18]. In Spain, as a result of all of the above, a regulatory framework was created that included the figure of the high-level athlete in the Sports Law of 1990 [19]. This meant that the athletes were guaranteed the necessary preparation and training to maintain their physical and technical fitness, as well as participation in official competitions for which they qualified [20]. This Law promoted the development of high-level and high-performance sports by establishing, among other measures, a public investment contribution through annual allocations included in the National Budget, which are still maintained to this day [19]. With the advent of this Law, Spain has been establishing the guidelines for high-level and high-performance athletes, for them to have sufficient and adequate human and material resources for their preparation [21]. At the same time, the Association of Olympic Athletes (ADO) program was created in 1987, with the objective of improving the performance of Spanish athletes in the Olympic Games of Barcelona ‘92 [19, 22]. Subsequently, Royal Decree 971/2007 of July 13, 2007 was published, which specified the scholarships or aid granted by the Consejo Superior de Deportes (CSD, Sports Council) to live and train at the High Performance Centers (CAR). Similarly, to complete the academic and professional training of these athletes, measures such as the reservation of university places in higher education for elite athletes were approved [19]. As a by-product of all this, in recent decades there has been a progressive professionalization, technification and economic dependence in the world of sport, which especially affects elite and high-performance sports [23].

However, professionalization may not have reached both genders equally in Spain. Until the Rio 2016 Olympic Games, out of the 3649 athletes who had represented Spain, 74.6% were men, who won 67.13% of Spain’s total Olympic medals. Not in vain, in the first six Olympic editions in which Spain officially participated (1920–1952), only two women qualified (both in Paris 1924, in tennis) without obtaining any medal, while men won six medals in that period. From Rome 1960 onwards, there was an incipient presence of women in the Spanish Olympic team without ever exceeding 15% of the total number of qualified athletes and without obtaining any medal either. Barcelona 1992 was the true explosion of elite women’s sport in Spain. Their participation reached 30% (125 out of 421 participants), winning 36% of the Spanish medals in those Olympic Games [23]. Nonetheless, since 2008, a change in this trend has been observed, as observed by the Spanish Olympic women reaching parity quotas of participation in the Olympic editions of Beijing, London, and Rio, having even surpassed the number of medals won by men in 2012 and 2016 [23, 24]. The historical performance of Spanish Olympic women must be put in context with the lack of equality for women’s sport within the Olympic movement. For example, women’s track and field events were not approved until 1928 or field hockey until 1984 [25], both disciplines in which Spanish women have won gold medals. As for the inequalities in participation, in 1920, Spain’s, first official OG, only 2.6% were women. By 1952, this percentage increased at 10.5%; in 1972 at 15%; and in Rio 2016, a total 45% of athletes were women [26].

Despite all of the above, no studies have been found that analyzed the probabilities of high-level athletes according to gender for accessing programs that promote the professionalization of sports, and participation and success in the OG in Spain. This could explain the gender differences in these parameters and the trend towards more egalitarian data in recent years. Therefore, the aim of this study was to analyze the probabilities of high-level Spanish athletes for participating and achieving sporting success in the 2008, 2012, and 2016 OG. For this, the variables of gender, having obtained a scholarship from the Association of Olympic Athletes (ADO) program and training in a High Performance Center (CAR), were analyzed.

## Materials and methods

### Study design

The design of this research was descriptive and cross-sectional. The STROBE statement [27] was followed for the development of the manuscript. Before the study began, approval was obtained from the institutional ethics committee (code 19.06.2015). In addition, the Spanish Olympic Games ceded the data collected to the Center for Olympic Studies of the Catholic University of Murcia, Spain, confidentially.

### Participants

A total of 3757 high-level athletes from Spain between 2005 and 2016 (2398 men and 1359 women) participated in the present study. The sample corresponded to the sample universe. The inclusion criteria were: 1) to be a high-level athlete in Spain between 2005 and 2016, with publication of one’s name in the Official State Bulletin and the Annual Report from the Sports Council of Spain.

### Procedure

For this study, data were collected on the participation of high-level athletes in the 2008, 2012 and 2016 Olympic Games; on the medals and diplomas obtained in these three Games, both in total and medals and diplomas separately, and for each type of metal; on participation in the finals in these Games; on obtaining an ADO scholarship; and on whether they trained at the CAR. All of these variables were collected, and scored using a dichotomous scale (yes or no) for each of the high-level athletes.

Medals are awarded for finishing in the top three of a competition. The type of metal indicates the position (1st gold, 2nd silver, and 3rd bronze). Diplomas are awarded for finishing in 4th to 8th place, inclusive. Both medalists and diploma holders are considered finalists. In both individual and team competitions, only one medal/diploma is awarded; while finalists are considered to be all the team members who have finished in the first eight positions.

The datasets generated for this study are available from the Zenodo database (DOI: 10.5281/zenodo.4663047).

### Statistical analysis

After analyzing the normality of the variables with a Kolmogorov-Smirnov test, a descriptive analysis was performed for the qualitative variables (counts and percentages). The Cramer´s V statistic was utilized for the post hoc comparison of 2x2 tables, and the contingency coefficient statistic was used for 2xn tables, showing the value of the statistic and the p value. The maximum expected value was 0.707; a low association was indicated if r<0.3; a moderate association if the r value was between 0.3 and 0.5, and a high association if r>0.5. The statistical analysis was performed using the statistical package SPSS 21.0 for Windows. An error of p≤0.05 was established.

## Results

Table 1 shows the gender differences of high-level athletes who participated in the 2008, 2012, and 2016 OG, trained at CARs, or obtained a scholarship from the ADO program. It was found that while there was a greater number of men considered as high-level athletes who participated in an OG, trained at a CAR, or obtained an ADO scholarship, among the women analyzes, it was statistically more likely for them to attend an OG, train at a CAR, or obtain a scholarship from the ADO program. The same trend was found when analyzing each of the three Olympic cycles separately, except for participation in the 2012 OG or CAR attendance in 2016; although the associations for all the variables were low.

**Table 1:** Differences between males and females in participation in an OG, CAR training, and ADO plan scholarships, in general and according to Olympic cycles.

**TOTAL (n = 3757)**				
**Variable**	**Category**	**Men (n = 2398)**	**Women (n = 1359)**	χ^2^; r*;p
Participation in OG	Yes	499 (20.8%)	376 (27.7%)	χ^2^ = 22,838; r* = 0,078; p = 0,000
	No	1899 (79.2%)	983 (72.3%)	
Training at CAR	Yes	651 (27.1%)	447 (33.9%)	χ^2^ = 13,847; r* = 0.061; p = 0,000
	No	1747 (72.9%)	912 (67.1%)	
ADO scholarship	Yes	332 (13.8%)	275 (20.2%)	χ^2^ = 26,151; r* = 0.083; p = 0,000
	No	2066 (86.2%)	1084 (79.8%)	
**2008 OLYMPIC CYCLE (n = 805)**				
**Variable**	**Category**	**Men (n = 521)**	**Women (n = 284)**	χ^2^; r*;p
Participation in OG	Yes	164 (31.5%)	121 (42.6%)	χ^2^ = 9.952; r* = 0.111; p = 0.00
	No	357 (68.5%)	163 (57.4%)	
Training at CAR	Yes	167 (32.1%)	117 (41.2%)	χ^2^ = 6.730; r* = 0.091; p = 0.009
	No	354 (67.9%)	167 (58.8%)	
ADO scholarship	Yes	126 (24.2%)	93 (32.7%)	χ^2^ = 6.804; r* = 0.092; p = 0.009
	No	395 (75.8%)	191 (67.3%)	
**2012 OLYMPIC CYCLE (n = 1324)**				
**Variable**	**Category**	**Men (n = 863)**	**Women (n = 461)**	χ^2^; r*;p
Participation in OG	Yes	170 (19.7%)	111 (24.1%)	χ^2^ = 3.447; r* = 0.051; p = 0.063
	No	693 (80.3%)	350 (75.9%)	
Training at CAR	Yes	231 (26.8%)	151 (32.8%)	χ^2^ = 5.248; r* = 0.063; p = 0.022
	No	632 (73.2%)	310 (67.2%)	
ADO scholarship	Yes	108 (12.5%)	91 (19.7%)	χ^2^ = 12.283; r* = 0.096; p = 0.000
	No	755 (87.5%)	370 (80.3%)	
**2016 OLYMPIC CYCLE (n = 1628)**				
**Variable**	**Category**	**Men (n = 1014)**	**Women (n = 614)**	χ^2^; r*;p
Participation in OG	Yes	165 (16.3%)	144 (23.5%)	χ^2^ = 12.823; r* = 0.089; p = 0.000
	No	849 (83.7%)	470 (76.5%)	
Training at CAR	Yes	253 (25.0%)	179 (29.2%)	χ^2^ = 3.465; r* = 0.046; p = 0.063
	No	761 (75.0%)	435 (70.8%)	
ADO scholarship	Yes	98 (9.7%)	91 (14.8%)	χ^2^ = 9.908; r* = 0.078; p = 0.002
	No	916 (90.3%)	523 (85.2%)	

OG: Olympic Games; CAR: High Performance Center; ADO: Association of Olympic Athletes

Table 2 shows the differences between male and female Olympians with respect to winning medals, diplomas, or being a finalist in the Olympic Games. In absolute numbers, men won a higher number of medals, diplomas, or reached the finals as compared to women. The same trend was found at Beijing 2008. In London 2012, women won more medals than men. Already in Rio 2016, the number of medals and finalists was higher for women, while the number of diplomas was higher for men. However, when analyzing the results of the chi-squared test, it was found that gender did not seem to significantly influence these aspects, except for the probability of being a finalist, where there was a significantly higher percentage of women. When analyzing the differences in the relationship of these variables according to the Olympic cycle, it was found that there were significant differences in London 2012, where women won more medals in general, and specifically bronze medals, than men; and in Rio 2016, where women were finalists in a higher percentage of medals. In all cases, the association was low.

**Table 2:** Differences between male and female Olympians in medals, diplomas and finalists overall and by Olympic cycles.

**TOTAL (n = 3757)**				
**Variable**	**Category**	**Men**	**Women**	χ^2^; r*;p
		**(n = 499)**	**(n = 376)**	
Medal or diploma	Yes	90 (18.0%)	68 (18.1%)	χ^2^ = 0,000; r* = 0.001; p = 0,985
	No	409 (82.0%)	308 (81.9%)	
Medal	Yes	28 (5.8%)	26 (6.9%)	χ^2^ = 0,629; r* = 0,027; p = 0,428
	No	471 (94.4%)	350 (93.1%)	
Gold	Yes	9 (1.8%)	7 (1.9%)	χ^2^ = 0,004; r* = 0.002; p = 0,949
	No	490 (98.2%)	369 (98.1%)	
Silver	Yes	13 (2.6%)	12 (3.2%)	χ^2^ = 0,273; r* = 0.018; p = 0,602
	No	486 (97.4%)	363 (96.8%)	
Bronze	Yes	6 (1.2%)	7 (1.9%)	χ^2^ = 0,645; r* = 0.027; p = 0,422
	No	493 (98.8%)	368 (98.1%)	
Diploma	Yes	62 (12.4%)	42 (11.2%)	χ^2^ = 0,322; r* = 0.019; p = 0,570
	No	437 (87.6%)	334 (88.8%)	
Finalists	Yes	255 (51.1%)	219 (58.2%)	χ^2^ = 4,406; r* = 0.071; p = 0,036
		244 (48.9%)	157 (41.8%)	
	No			
**2008 OLYMPIC CYCLE (n = 285)**				
**Variable**	**Category**	**Men (n = 164)**	**Women (n = 121)**	χ^2^; r*;p
Medal or diploma	Yes	35 (21.3%)	19 (15.7%)	χ^2^ = 1.442; r* = 0.071; p = 0.230
	No	129 (78.7%)	102 (84.3%)	
Medal	Yes	14 (8.5%)	5 (4.1%)	χ^2^ = 2.171; r* = 0.087; p = 0.141
	No	150 (91.5%)	116 (95.9%)	
Gold	Yes	5 (3.0%)	0 (0.0%)	χ^2^ = 3.755; r* = 0.115; p = 0.053
	No	159 (97.0%)	121 (100.0%)	
Silver	Yes	7 (4.3%)	4 (3.3%)	χ^2^ = 0.174; r* = 0.025; p = 0.677
	No	157 (95.7%)	117 (96.7%)	
Bronze	Yes	2 (1.2%)	1 (0.8%)	χ^2^ = 0.103; r* = 0.019; p = 0.748
	No	162 (98.8%)	120 (99.2%)	
Diploma	Yes	21 (12.8%)	14 (11.6%)	χ^2^ = 0.099; r* = 0.019; p = 0.754
	No	143 (87.2%)	107 (88.4%)	
Finalists	Yes	96 (58.5%)	58 (47.9%)	χ^2^ = 3.151; r* = 0.105; p = 0.076
	No	68 (41.5%)	63 (52.1%)	
**2012 OLYMPIC CYCLE (n = 281)**				
**Variable**	**Category**	**Men (n = 170)**	**Women (n = 111)**	χ^2^; r*;p
Medal or diploma	Yes	27 (15.9%)	21 (18.9%)	χ^2^ = 0.437; r* = 0.039; p = 0.508
	No	143 (84.1%)	90 (81.1%)	
Medal	Yes	6 (3.5%)	12 (10.8%)	χ^2^ = 5.939; r* = 0.145; p = 0.015
	No	164 (96.5%)	99 (89.2%)	
Gold	Yes	1 (0.6%)	3 (2.7%)	χ^2^ = 2.140; r* = 0.087; p = 0.144
	No	169 (99.4%)	108 (97.3%)	
Silver	Yes	5 (2.9%)	5 (4.5%)	χ^2^ = 0.478; r* = 0.041; p = 0.489
	No	165 (97.1%)	106 (95.5%)	
Bronze	Yes	0 (0.0%)	4 (3.6%)	χ^2^ = 6.215; r* = 0.149; p = 0.013
	No	170 (100.0%)	96.4 (%)	
Diploma	Yes	21 (12.4%)	9 (8.1%)	χ^2^ = 1.269; r* = 0.067; p = 0.260
	No	149 (87.6%)	102 (91.9%)	
Finalists	Yes	83 (48.8%)	62 (55.9%)	χ^2^ = 1.330; r* = 0.069; p = 0.249
	No	87 (51.2%)	49 (44.1%)	
**2016 OLYMPIC CYCLE (n = 309)**				
**Variable**	**Category**	**Men (n = 165)**	**Women (n = 144)**	χ^2^; r*;p
Medal or diploma	Yes	28 (17.0%)	28 (19.4%)	0.317; r* = 0.032; p = 0.573
	No	137 (83.0%)	116 (80.6%)	
Medal	Yes	8 (4.8%)	9 (6.3%)	0.291; r* = 0.031; p = 0.590
	No	157 (95.2%)	135 (93.8%)	
Gold	Yes	3 (1.8%)	4 (2.8%)	0.320; r* = 0.032; p = 0.572
	No	162 (98.2%)	140 (97.2%)	
Silver	Yes	1 (0.6%)	3 (2.1%)	1.330; r* = 0.066; p = 0.249
	No	164 (99.4%)	140 (97.9%)	
Bronze	Yes	4 (2.4%)	2 (1.4%)	0.422; r* = 0.037; p = 0.516
	No	161 (97.6%)	141 (98.6%)	
Diploma	Yes	20 (12.1%)	19 (13.2%)	0.080; r* = 0.016; p = 0.777
	No	145 (87.9%)	125 (86.8%)	
Finalists	Yes	76 (46.1%)	99 (68.8%)	16.118; r* = 0.228; p = 0.000
	No	89 (53.9%)	45 (31.3%)	

## Discussion

The objective of the present research was to analyze the probabilities of Spanish high-level athletes in relation to gender, for being granted with an ADO program scholarship, training at a CAR, and/or participating and achieving sporting success in the 2008, 2012, and 2016 Olympic Games. The main finding was that women who were high-level athletes were more likely to obtain an ADO scholarship, train at a CAR, and qualify for the OG, than men. The latter may be a consequence of the two previous findings, since obtaining an ADO scholarship allows them to dedicate themselves professionally to sports [22, 24], while training at a CAR guarantees first level human, technical, and logistical resources for training [19, 28]. Both factors promote the optimization of sports performance. On the other hand, the higher percentage of high-level female athletes who obtained a scholarship from the ADO plan or trained at CAR, could be a result of the government policies related to the improvement of the role of women in Spanish society instituted in recent years, which affected the field of sports [29–31]. In this regard, complementary support programs in Spain such as the "Women and Sport" initiative from the Sports Council (2007) [30], the Plan for Equal Opportunities between Women and Men of the Ministry of Labor and Social Affairs (2003–2006) [32], the Organic Law 3/2007 of March for the effective equality between men and women (2007) [33], the Equal Opportunity Strategic Plan (2008–2011) [34], the II Plan for equality between women and men in the General State Administration and its Public Bodies (2015) [35], or the annual calls for applications for assistance to women, which provide special coverage for mothers and student athletes [30], all of which may have provided an additional stimulus for the increase in women’s sports performance. The entry of women into high-performance sports with the support of stakeholders has not only provided women with the opportunity to demonstrate their sporting competence, but as they have achieved sporting successes, the media coverage generated by these successes reinforced their role as sportswomen and women athletes [36, 37]. Comparing the results of the present research with previous studies, different authors have pointed out that the egalitarian demand of women in the field of sports has resulted in the entry of women into all sports structures [38, 39], although women’s access to sports management positions may still be somewhat limited [29].

Another important finding of the present research was that Spanish female Olympic athletes showed a higher probability for being finalists than male athletes in general, in particular in Rio 2016, and a higher probability for obtaining medals, especially bronze medals, in London 2012. This fact is especially significant when as Zheng et al. [40] have shown that from Barcelona 1992 to Rio 2016, the analysis of female competitions suggests that competitive balance has remained largely unchanged. Based on the results of the present research, it seems that despite women entering high-level sports later, they have done so with more strength, showing an upward trend in the achievement of great sporting success in the OG, as previous research has shown [37]. This could be due to equal access to official recognition as a high-level athlete, and to all the structures promoted by the stakeholders to favor professionalization in sports [19, 24]. This may have given Spanish athletes a competitive advantage over those from other countries without such support networks [31, 41]. Another possible reason could be that changes in aspects such as the socio-economic status of the female athlete and society’s attitudes towards gender issues in general, have influenced the Olympic success of female athletes [41]. In this sense, Spanish women have been acquiring prominence and relevance in the world of sports in recent decades, in parallel with their active incorporation into all spheres of society [30, 42]. However, future studies are needed to further analyze this trend in future OG.

Despite the achievements in women’s sports, the data from the present investigation indicate that in absolute terms, there is still a greater number of men who are considered high-level athletes in Spain, as well as ADO scholarship holders, training in a CAR, who attend OGs; who in general terms have achieved successful results in the OGs as compared to women. Previous studies have reported similar trends [43]. Gender differences in these areas may be due to the historical existence of a greater number of barriers for women seeking to achieve the status of professional athletes as compared to their male counterparts [44–46]. An example of this is that the eligibility criteria for the OG have traditionally been more restrictive for women, which has led to unequal opportunities for female athletes, not only for participating in the OG, but also in the access to technification and professionalization programs [47–49]. Although this matter has been evolving in recent years, there is still inequality in this area. Therefore, it seems necessary to modify the sports modalities that do not offer an equitable program for men and women, to eliminate the differences that grant more privileges to male events over female ones [48]. In this respect, it has been found that sports federations and other sports organizations should promote gender policies and create *ad hoc* programs to promote equality in all areas of sports [29, 50]. In agreement with this, previous research studies have pointed out the importance of the social context and the need for stakeholder support to enable the holistic development of the female athlete [41, 45], which allows not only their growth in sports, but also their educational progress with their inclusion in dual career programs [51, 52], and the resulting employment success during and after their active careers [53, 54]. These factors are essential for achieving the complete disappearance of the gender gap in sports [41, 53].

With respect to the limitations of the present study, the lack of data prior to 2008 for the analysis of longer-term developments, is worth noting. In addition, as most of the programs designed to eliminate the gender gap in sports are relatively new, there are no data on their ulterior impacts. These aspects need to be addressed in future research.

## Conclusions

A higher percentage of high-level female athletes in Spain have access to high-level athlete support programs such as ADO and CAR. In addition, there is also a greater participation in the OG, and in recent years, there has been a tendency to achieve great sporting successes in these events. However, in absolute terms, the number of male Spanish athletes who consider themselves to be high-level athletes, who access athlete support programs, and who participate in the OG is still slightly higher.
